# Competition and Opportunity Shape the Reproductive Tactics of Males in the Ant *Cardiocondyla obscurior*

**DOI:** 10.1371/journal.pone.0017323

**Published:** 2011-03-29

**Authors:** Sylvia Cremer, Alexandra Schrempf, Jürgen Heinze

**Affiliations:** Evolution, Behaviour and Genetics, Biology I, University of Regensburg, Regensburg, Germany; University of Sheffield, United Kingdom

## Abstract

Context-dependent adjustment of mating tactics can drastically increase the mating success of behaviourally flexible animals. We used the ant *Cardiocondyla obscurior* as a model system to study adaptive adjustment of male mating tactics. This species shows a male diphenism of wingless fighter males and peaceful winged males. Whereas the wingless males stay and exclusively mate in the maternal colony, the mating behaviour of winged males is plastic. They copulate with female sexuals in their natal nests early in life but later disperse in search for sexuals outside. In this study, we observed the nest-leaving behaviour of winged males under different conditions and found that they adaptively adjust the timing of their dispersal to the availability of mating partners, as well as the presence, and even the type of competitors in their natal nests. In colonies with virgin female queens winged males stayed longest when they were the only male in the nest. They left earlier when mating partners were not available or when other males were present. In the presence of wingless, locally mating fighter males, winged males dispersed earlier than in the presence of docile, winged competitors. This suggests that *C. obscurior* males are capable of estimating their local breeding chances and adaptively adjust their dispersal behaviour in both an opportunistic and a risk-sensitive way, thus showing hitherto unknown behavioural plasticity in social insect males.

## Introduction

Conspecific males of many animal species employ different ways to increase their reproductive success. Such alternative reproductive tactics are easily recognized when associated with pronounced morphological differences, e.g., when territorial males have well-developed weapons while sneakers or female mimics have not [1], [2]. In addition, morphologically uniform males sometimes adjust their breeding behaviour to changes in their social and ecological environment. For example, individuals may choose between philopatry and dispersal based on the local presence of mating partners and competitors, as occurs in some vertebrate species [1], [3]–[5]. It is so far not clear whether also males of social insects are able to perform such adaptive mating decisions by behavioural plasticity.

Social insects (the social bees and wasps, ants and termites) typically mate within a swarm flight, in which scramble competition is the dominant form of male-male competition [6]. However, in some species, more complex sexual behaviour has evolved. Among these is our study system, the ant species *Cardiocondyla obscurior* that is characterized by a high social plasticity. First, colonies of this species can have variable queen numbers from a single to multiple queens and second, two male morphs co-occur and form a male diphenism with wingless fighter males (“ergatoid males”) and peaceful winged males. Wingless males engage in lethal combat for access to female nestmates, whereas winged males resemble the typical docile ant male in behaviour and physiology [7]–[11]. Wingless males stay lifelong in their natal nests, but the behaviour of winged males is more flexible: they mate within the nest early during their adult lives, but later disperse and search for female sexuals outside [7], [12], [13].

Here, we investigated whether winged males of *C. obscurior* can adaptively adjust the timing of their dispersal from the natal nest to local mating opportunities. We indeed observed that they stay longer in their natal nests when mating partners are available but leave earlier when male competitors are present. We also found that winged males adjust their departure time to the male morph with which they share the nest. They leave much earlier in the presence of a wingless fighter male than of a second peaceful winged male, revealing that the dispersal decision of winged males is also dependent on the type of male-male competition.

## Methods

We collected 45 *Cardiocondyla obscurior* colonies from their nests in folded leaves in an experimental lemon plantation in Una, Bahia, Brazil. Collecting of colonies was allowed by Brazilian authorities (permit RMX 004/02). The experiments comply with the laws of Germany.

Ants were reared in the laboratory at a 30°/25°C day/night cycle in three-chamber plastic boxes with a plaster floor [14]. One compartment contained a cavity in the ground covered with a microscope slide, where the ants formed their nests. Food (diluted honey and pieces of cockroaches) was offered in a second compartment, and a third contained a water resource. The three compartments were connected by small holes (0.5 cm in diameter), which allowed free moving of all castes between the different parts.

Behavioural observations were performed in small subcolonies obtained from these stock colonies, each containing one reproductive queen, 10–20 workers, 10–15 brood items (larvae, pupae), and a varying number of sexuals: female sexuals (i.e. virgin young queens), wingless males and winged males; as specified for each experiment. We first investigated when winged males leave a nest containing several (5–7) virgin female sexuals when they are a) the only male in the colony (n = 10 replicates), b) share it with another winged male (n = 9), or c) share it with an aggressive ergatoid male (n = 9). In each experimental nest, the focal winged male was present as a pigmented pupa, i.e. one or two days prior to emergence, while the other males were already present as 1–2 day old adults when the focal male eclosed. To differentiate between the two winged males (setup b), we clipped the wings of the non-focal winged male (scissors B-41, Bioform). After the winged male had emerged, its location inside the nest box was checked daily. The first instance during which focal winged males were observed to move to the food or water compartment was defined as the time of dispersal. In all cases, the non-focal males (be it winged or wingless) had not left the nest at the time of nest leaving of the focal males. Previous studies had revealed that once a winged male has left the nest compartment it never returned into it. When experimentally placed back into the nest it either left again within several hours or was killed by the workers of the colony (S.C., unpublished data). The behaviour that wingless males would show to their winged nest males after their switch-point towards nest-leaving can therefore not directly be observed.

To determine the effect of female sexuals on the timing of dispersal we added two treatments without female sexuals, i.e., experimental colonies contained only an old reproductive queen, workers and brood, and either a wingless male in addition to the focal winged male (n = 12), or no other male (n = 17). Unfortunately, due to the limited number of winged males we could not set up additional setups without female sexuals but with two winged males.

Independence of data points within the five groups was assured by using different source colonies as donors for each replicate. Statistical tests for independent sampling were applied to test for differences between groups as we used a different subcolony for each observed winged male (and did not re-use existing subcolonies). We performed a One Way ANOVA to test for the difference between dispersal time of winged males when alone, with another winged male, or a wingless male, and a Two Way ANOVA to compare the effect of the presence or absence of both a wingless male or female mating partners, as well as their interaction. Pairwise comparisons between groups were performed by a posthoc SNK test. Normality and equal variance tests were passed in both analyses. As we re-used two groups (winged male alone and winged plus wingless male in the presence of female sexuals) in both analyses, we performed a Bonferroni correction and adjusted our significance level α to 0.025 for both analyses. All statistical analyses were performed with Sigma Stat 2.03.

## Results

**Figure 1:**
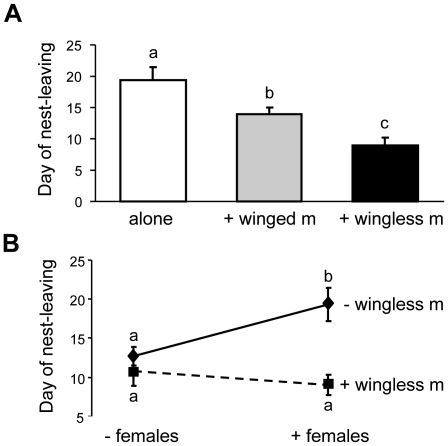
Dispersal time of winged *Cardiocondyla* males from their natal nest. **A**) In the presence of virgin queens, winged males stayed longer in the colony when being the single male in the colony (white) than when another male was present. They emigrated earlier when the second male was a wingless fighter male (black) than when it was a peaceful winged male (grey; mean ± s.d., significance groups marked with different letters). **B**) In the presence of a wingless fighter male, winged males always left the nest early. They prolonged their stay in nests without a wingless male only when virgin female sexuals were present (mean ± s.d., significance groups marked with different letters).

Winged males left colonies with virgin female sexuals 19.3±6.7 (mean ± s.d.) days after emergence when they were the only male in the nest. They left the nest approximately five days earlier (14.0±3.2 days) when sharing it with a second winged male and yet another five days earlier (9.0±3.8 days) when they competed with a wingless male (Fig. 1A; ANOVA, *F*_2,25_ = 10.39, *P*<0.001; pairwise SNK posthoc tests: all significant). As reported previously, winged males readily mated with the female sexuals and achieved a similar copulation frequency as wingless males [13].

In the absence of female sexuals, winged males left the nest after 12.7±5.0 days when they were the only male in the colony and after 10.8±6.5 days when they shared the nest with a wingless male. When comparing the timing of dispersal between colonies with and without female sexuals, we found that the presence of female sexuals delays dispersal by winged males only if no wingless males are present (Fig. 1B). This is indicated by the significant interaction term in a Two Way ANOVA (effect of presence/absence of wingless males: *F*_1,47_ = 13.29, *P*<0.001; effect of presence/absence of female sexuals: *F*_1,47_ = 2.15, *P* = 0.15; interaction wingless males and female sexuals: *F*_1,47_ = 6.31, *P*<0.016). The effect of female mating partners on the dispersal of winged males is thus context-dependent.

We did not observe aggressive interactions among males of either morph or between males and workers or queens, suggesting that males independently adjust the timing of dispersal in response to their social environment.

## Discussion

Our data show that winged males of *Cardiocondyla obscurior* adjust their reproductive behaviour to mating opportunities and the presence and type of male competitors. Winged males left the nest later when they were the only male in a colony than when other males were present. They stayed significantly longer in the natal nest in the presence of another docile winged male than in the presence of a wingless fighter male. This reflects different degrees of male-male competition. Whereas winged competitors only decrease the availability of virgin queens and, if female sexuals mated multiply, potentially increasing sperm competition, the presence of wingless males introduces the risk of being killed or injured. Winged males chemically mimic female sexuals during the first few days of their adult lives and therefore are relatively safe against lethal attacks from wingless males [13], [15]. However, their chemical female mimicry becomes less effective with age and the chemical profile of ten day old winged males does no longer overlap with that of virgin females [13]. Interestingly, we here found that winged males that share a nest with an aggressive wingless male leave already one day earlier, namely on average at the age of nine days. In contrast, winged males stay much longer in the nest if being the only male in a colony or if they share it with another peaceful winged male. This suggests that winged males ‘may be aware’ of the loss of their chemical protection against wingless males when they grow older and therefore leave the nest to prevent possible attacks, which are predicted by theory [12].

Over all, the reproductive behaviour of winged males of *C. obscurior* is surprisingly flexible. They appear to be capable of estimating their breeding chances in the natal nest in respect of the presence of competitors and the number of potential mating partners and adjust their dispersal behaviour accordingly. The behavioural plasticity shown by winged *Cardiocondyla* males allows context-dependent choices that resemble the often complex decision-making about staying or leaving in foraging animals [16] and the opportunistic breeding tactics known from vertebrate species [1], [3].
